# Chitosan Grafted With β-Cyclodextrin: Synthesis, Characterization, Antimicrobial Activity, and Role as Absorbefacient and Solubilizer

**DOI:** 10.3389/fchem.2018.00657

**Published:** 2019-01-10

**Authors:** Wen-Ya Ding, Si-Di Zheng, Yue Qin, Fei Yu, Jing-Wen Bai, Wen-Qiang Cui, Tao Yu, Xing-Ru Chen, God'spower Bello-Onaghise, Yan-Hua Li

**Affiliations:** ^1^College of Veterinary Medicine, Northeast Agricultural University, Harbin, China; ^2^Heilongjiang Key Laboratory for Animal Disease Control and Pharmaceutical Development, Harbin, China; ^3^Northeastern Science Inspection Station, China Ministry of Agriculture Key Laboratory of Animal Pathogen Biology, Northeast Agricultural University, Harbin, China; ^4^College of Science, Northeast Agricultural University, Harbin, China

**Keywords:** chitosan grafted with β-cyclodextrin, antimicrobial activity, membrane integrity, uptake, solubility

## Abstract

We synthesized chitosan grafted with β-cyclodextrin (CD-g-CS) from mono-6-deoxy-6-(p-toluenesulfonyl)-β-cyclodextrin and chitosan. Two different amounts of immobilized β-cyclodextrin (β-CD) on CD-g-CS (Q_CD_: 0.643 × 10^3^ and 0.6 × 10^2^ μmol/g) were investigated. The results showed that the amino contents of CD-g-CS with Q_CD_ = 0.643 × 10^3^ and 0.6 × 10^2^ μmol/g were 6.34 ± 0.072 and 9.41 ± 0.055%, respectively. Agar diffusion bioassay revealed that CD-g-CS (Q_CD_ = 0.6 × 10^2^ μmol/g) was more active against *Staphylococcus xylosus* and *Escherichia coli* than CD-g-CS (Q_CD_ = 0.643 × 10^3^ μmol/g). Cell membrane integrity tests and scanning electron microscopy observation revealed that the antimicrobial activity of CD-g-CS was attributed to membrane disruption and cell lysis. Uptake tests showed that CD-g-CS promoted the uptake of doxorubicin hydrochloride by *S. xylosus*, particularly for CD-g-CS with Q_CD_ = 0.6 × 10^2^ μmol/g, and the effect was concentration dependent. CD-g-CS (Q_CD_ = 0.6 × 10^2^ and 0.643 × 10^3^ μmol/g) also improved the aqueous solubilities of sulfadiazine, sulfamonomethoxine, and sulfamethoxazole. These findings provide a clear understanding of CD-g-CS and are of great importance for reducing the dosage of antibiotics and antibiotic residues in animal-derived foods. The results also provide a reliable, direct, and scientific theoretical basis for its wide application in the livestock industry.

## Introduction

As a natural weak cationic polysaccharide, chitosan (CS) is made up of randomly distributed β-1,4-linked D-glucosamine and N-acetyl-D-glucosamine (Song et al., 2017). The ubiquity, non-toxicity and biodegradability of CS (Li et al., 2016) has made it useful in various scientific fields for many years (Guo et al., 2016). The unique characteristics of CS have prompted investigators (Castro Domingues et al., 2012) to propose CS as an alternative adsorbent. CS is also reported to have some anti-inflammatory activity (Maeda and Kimura, 2004). As a slow-release drug carrier, CS nanoparticles enhance the intestinal absorption of insulin (Pan et al., 2002). In addition, CS can inhibit the growth of pathogens as an antimicrobial agent (Badawy et al., 2014). CS was previously reported to significantly inhibit the growth of bacteria (No et al., 2002). CS and its derivatives have also been extensively studied as absorption enhancers. N-trimethyl CS chloride was reported to significantly increase the oral absorption of buserelin and octreotide (van der Merwe et al., 2004).

Cyclodextrins are cyclic oligosaccharides made up of different numbers of glucose molecules that are joined together by α-1,4 bonds. Cyclodextrins are grouped into α-, β-, and γ-cyclodextrins. The most popular and widely studied member of the group is the β-cyclodextrin (β-CD) because it is a common host molecule for a variety of aqueous species due its availability and ideal molecular size (Cho et al., 2014). Furthermore, the outer layer of β-CD is hydrophilic in nature, while the center cavity is hydrophobic. The hollow part of the central cavity is lipophilic in nature and can contain many hydrophobic guest molecules based on hydrophobic interactions. According to Szejtli (2004), the formation of inclusion complexes with cyclodextrins can significantly increase the aqueous solubility of poorly soluble drugs. A good example is the formation of cyclodextrin inclusion complexes with natamycin (Koontz and Marcy, 2003).

Bacterial infection is a major challenge in the livestock industry. Farmers depend on antibiotics to prevent and control bacteria-related infections, and antibiotics have thus played a major role in the development of the industry (Long et al., 1990). However, many factors limit the clinical application of antibiotics. For instance, the long-term or heavy use of antibiotics frequently causes bacterial resistance and results in antibiotic residues in animal-derived foods, which can harm human health (de Jong et al., 2018; Todorovic et al., 2018; Yan et al., 2018). In addition, some antibiotics that are difficult to dissolve in water cannot be used in practical applications because the most important physicochemical property of a drug is its ability to be dissolved in water, thus, compounds that are not soluble in water are hardly used as drugs (Hanaee et al., 2005). To improve the efficacy of antibiotics, reduce the antibiotic dosage, overcome bacterial resistance and increase the solubility of antibiotics, it is of great importance to develop an excipient that can inhibit bacteria, reduce antibiotic use, and increase the solubility of antibiotics in water. CS can be used as antimicrobial agent to inhibit bacterial growth (Badawy et al., 2014) and as an absorption enhancer to promote drug oral absorption (van der Merwe et al., 2004). β-CD can increase the aqueous solubility of poorly soluble drugs (Szejtli, 2004). Based on this, we hypothesize that chitosan grafted with β-cyclodextrin (CD-g-CS) will function as a unique excipient with double potency. However, there are few reports on the antibacterial activity of CD-g-CS. In addition, no one has reported the mechanism of the antibacterial activity of CD-g-CS or the effects of CD-g-CS on drug uptake and the solubilization of insoluble drugs. Most existing studies focused on the application of CD-g-CS as an adsorption agent (Sajomsang et al., 2012), scaffold for tissue engineering applications (Prabaharan and Jayakumar, 2009), and compound for controlled drug release (Chen et al., 2017). Thus, the above properties of CD-g-CS were studied in this paper.

In this study, we used Fourier transform infrared spectroscopy (FT-IR) and ^1^H nuclear magnetic resonance spectroscopy (^1^H NMR) to characterize the structure of CD-g-CS. Acid–base titration was used to determine the amino content of CD-g-CS. Thereafter, the antimicrobial activity of CD-g-CS was evaluated. At the same time, ultrastructural analysis and cell membrane integrity tests revealed the complex mechanism of CD-g-CS activity against *Escherichia coli* and *Staphylococcus xylosus*. To demonstrate the ability of CD-g-CS to promote drug uptake, an uptake test was conducted. We also investigated the effect of CD-g-CS on the solubilization of antibiotics that are difficult to dissolve in water. This study is of great importance for improving the efficacy of antibiotics, reducing the dosage of antibiotics, and reducing antibiotic residues in animal-derived foods. The findings related to CD-g-CS also provide new ideas for addressing bacterial resistance in the cultivation industry.

## Materials and Methods

### Materials

Chitosan, having a molar mass of 150 kDa and a deacetylation degree (DD) of over 90%, was purchased from Beijing Biotopped Science & Technology Co. Ltd (China) while Sinopharm Chemical Reagent Co. Ltd (China) supplied the β-Cyclodextrin (β-CD). P-Toluenesulfonyl chloride was purchased from Shanghai Macklin Biochemical Co. Ltd (China). Sulfadiazine, sulfamonomethoxine sulfamethoxazole, and doxorubicin hydrochloride were purchased from Shanghai Yuan Ye Biochemical Co. Ltd (China). Acetic acid, calcium, dimethyl sulfoxide (DMSO), acetone, methyl orange, and sulfuric acid were purchased from Tianjin Kermel Analytical Reagent Co. Ltd (China). Trypticase Soy broth (TSB) was purchased from Summus Ltd (China). Super Optimal broth with Catabolite repression (Soc Medium) was purchased from Sijiqing Ltd., (China).

### Preparation of Mono-6-Deoxy-6-(p-Toluenesulfonyl)-β-Cyclodextrin (6-OTs-β-CD)

β-CD (2.5 g) was suspended in 6.5 mL of DMSO, and 3.75 g of p-toluenesulfonyl chloride in 6 mL of DMSO was added dropwise. The solution was stirred for 24 h at 45°C, and 30 mL of acetone was added by gradual droppings. The solution immediately crystallized, and a white precipitate was observed. This white precipitate was filtered, rinsed with precooled acetone. This procedure was repeated three times.

### Preparation of CD-g-CS

CD-g-CS was synthesized using a modified procedure derived from Gonil (Gonil et al., 2011). CS (0.25 g) was dissolved in 1% (*v/v*) acetic acid. The 6-OTs-β-CD produced above was added into the CS/acetic acid solution. The set up was then stirred for 48 h at 45°C, and the solution was dialyzed for 7 d using deionized water. Finally, the solution was freeze-dried.

### Characterization of CD-g-CS

This procedure followed that of Gonil et al. (2011). The ^1^H NMR readings of the samples were recorded on a Bruker DPX300 spectrometer using tetramethylsilane as an internal standard and 1% DCl as a solvent at 25°C.

This procedure followed that of Zhu et al. (2006). The FT-IR scores of the products were recorded on a double-beam Mattson Galaxy Series FTIR-3000 spectrometer in the range of 4,000–600 cm^−1^ using KBr pellets.

### Measurement of the Apparent Amount of β-CD Immobilized Onto CS

The apparent amount of β-CDs immobilized on CS was determined using Yi's method (Yi et al., 2006) with slight modification. A standard curve was plotted in line with that of Yi et al. (2006) using the following procedure. We hydrolyzed 10 mg of CD-g-CS in 30 mL of sulfuric acid (0.5 mol/L) and stirred the solution for 10 h at 100°C. The resultant solution was poured into a measuring flask and diluted to 50 mL. A photospectrometer was used to determine the glucose content of the solution at a wavelength of 490 nm. The apparent amount of immobilized β-CD (Q_CD_) was calculated using Equation (1):

(1) $$\begin{array}{r} {\text{Q}}_{\text{CD}}=\frac{C\times 50\times 1000}{180\times 7\times W} \end{array}$$

where *C* is the concentration of glucose (μg/mL), *W* is the weight of CD-g-CS (mg), 50 is the final volume of CD-g-CS hydrolysate, 1,000 is the conversion factor that is used to convert CD-g-CS from mg to g (1 mg = 10^−3^ g), 180 is the molecular weight of glucose, and 7 is the number of units containing glucose groups in β-CD.

### Determination of the Amino Content of CD-g-CS

The amino content of CD-g-CS was determined as described by Hamdi et al. (2018) with a few modifications. CD-g-CS (30 mg) was dissolved in 5 mL of HCl (0.1 mol/L). The dissolution time was 1 h, and the setup was kept at room temperature. Methyl orange served as an indicator for the titration experiment. The acid was titrated against NaOH (0.1 mol/L). A sharp change in color change (from red to yellow) indicated the end point of the titration. This color change was followed by the appearance of suspended solutes in the solution. The volume of NaOH used and recorded. The amino content of CD-g-CS was calculated using the following Equation (2):

(2) $$\begin{array}{r} NH_{2}\%=\frac{\left(C_{1}V_{1}-C_{2}V_{2}\right)\times 0.016}{G}\times 100\% \end{array}$$

where 0.016 is the amount of amino acid equivalent to 1 mol/L HCl, *V*_1_ and *V*_2_are the volumes (mL) of HCl and NaOH solutions used, respectively; *C*_1_ and *C*_2_ are the concentrations (mol/L) of HCl and NaOH, respectively; and *G* is the weight of CD-g-CS (g).

### Antimicrobial Assessments

#### Bacterial Strains and Growth Conditions

The culturing of bacteria was done in line with the procedure described in Yang et al. (2015) with a few modifications. *S. xylosus* ATCC 700404 and *E. coli* ATCC 25922 were grown in TSB and SOC, respectively. All strains were subjected to aerobic incubation at 37°C for 16 h. The incubated strains were again subcultured under the same conditions. Cultured *S. xylosus* and *E. coli* were grown on TSB and SOC agar, respectively, and incubated at 37°C. Overnight cultures of *S. xylosus* and *E. coli* were diluted in sterile saline to produce a solution with a concentration of 1 × 10^8^ CFU/mL. The cultures of *S. xylosus* and *E. coli* were then diluted to a ratio of 1:1,000 in a TSB and SOC agar, respectively, at 48°C and used as inoculated layers.

#### Agar Diffusion Bioassay

Agar diffusion bioassay was performed according to Schmidt's method (Schmidt et al., 2009). For the base layer agar, 20 mL of TSB or SOC agar culture medium was poured into microbiological plates. After solidification, 5 mL of inoculated TSB or SOC agar medium was added to the base layer. In each plate, six stainless-steel cylinders were placed on the surface of the inoculated medium. Subsequently, 240 μL of 10, 5, or 2.5 mg/mL CD-g-CS was poured into each cylinder and filled to the mark. The plates were incubated for 16 h at 37°C. Finally, an electronic caliper was used to measure the diameters of the inhibition zones.

### Effect of CD-g-CS on Bacterial Membrane Integrity

Experiments were conducted as stated in Lou et al. (2011) with a few modifications. The cultured *S. xylosus* and *E. coli* were harvested at their logarithmic phase, washed and re-suspended in a sterile 0.9% saline solution. The bacterial suspensions were adjusted to an optical density (OD) at 630 nm of 0.5 ± 0.02. CD-g-CS solution was mixed with the bacterial suspension to give a final CD-g-CS concentration of 10 mg/mL. The mixture was then incubated at 37°C for 0, 60, 120, 240, 360, or 480 min. The mixture was centrifuged, and the supernatant was filtrated using a 0.22-μm filter membrane. The filtrates were analyzed using a microplate reader at 260 nm.

### Effect of CD-g-CS on Microcosmic Morphology of Bacteria

Acetic acid [1% (*v/v*)] was used to dissolve the CD-g-CS. The dissolved CD-g-CS solution was then adjusted to a pH of 5 using NaOH (8 mol/L). *S. xylosus* and *E. coli* in their logarithmic phase were harvested and re-suspended in solutions with and without 10 mg/mL CD-g-CS. Subsequently, the mixtures were incubated at 37°C for 1.5 h. Then the mixtures were incubated in fixation buffer [2.5 % (*w/v*) glutaraldehyde, pH 7.2] for 1.5 h at 4°C, and washed three times with 0.1 M cacodylate buffer (pH 7.0) for 20 min each time. The samples were dehydrated using a graded series of ethanol (50, 70, 95, and 100%). After the dehydrated samples were subjected to critical point drying and gold sputtering, they were examined by scanning electron microscopy (SEM) (Yang et al., 2015).

### Study on the Uptake of Drugs Promoted by CD-g-CS in *S. xylosus*

The cultured *S. xylosus* in the logarithmic phase were mixed with CD-g-CS (5, 2, or 0.5 mg/mL) containing 50 μmol/L doxorubicin hydrochloride. The mixtures were incubated at 37°C for 1 h. Subsequently, the *S. xylosus* solutions were centrifuged, washed three times with PBS (pH = 7.2), and resuspended in 500 μL PBS. For each sample, *S. xylosus* was sorted using a Flow Cytometry System (FCS). *S. xylosus* treated with only doxorubicin hydrochloride served as a control.

Cultured *S. xylosus* in the logarithmic phase was mixed with 2 mg/mL CD-g-CS containing 50 μmol/L doxorubicin hydrochloride. The mixtures were incubated at 37°C for 30 min, 1 or 3 h. The mixtures of *S. xylosus* were then centrifuged, washed three times, and re-suspended in 500 μL PBS (pH = 7.2). Each sample with *S. xylosus* was placed on a glass slide and observed by confocal laser scanning microscopy (CLSM). *S. xylosus* treated with only doxorubicin hydrochloride for 30 min, 1 or 3 h served as the control.

### The Effect of CD-g-CS on the Solubilization of Sulfonamides

#### Measurement of the Aqueous Solubility of Sulfadiazine, Sulfamonomethoxine, and Sulfamethoxazole

Aqueous solubility measurements were conducted according to the method of Wang and Wang (2000). Large amounts of sulfadiazine, sulfamonomethoxine, and sulfamethoxazole were added to distilled water in an equilibrium cell. The mixture was agitated until reaching equilibrium with water in the equilibrium cell and then kept for 30 min. A known amount of the upper layer of the mixture was removed from the equilibrium cell and diluted with distilled water. The calibration curve established by ultraviolet–visible spectrophotometry (UV–Vis) was used to determine the concentration of the solution.

#### Solubilization of Sulfadiazine, Sulfamonomethoxine, and Sulfamethoxazole With CD-g-CS

Solubilization was evaluated using the method described by Furuishi et al. (2017) with a few modifications. Acetic acid [1 mL, 1 % (*v/v*)] was used to dissolve 10 mg of CD-g-CS, and the pH of the solution was adjusted to be similar to that of the distilled water. Aliquots of sulfadiazine, sulfamonomethoxine, and sulfamethoxazole were separately added to 1 mL of 10 mg/mL CD-g-CS. A vortex mixer was used to mix the suspension followed by sonication for 20 min. The mixture was then stirred for 2 h at 25°C followed by the centrifugation of the suspension at 8,000 r/min for 10 min. The supernatant was filtered using a membrane with 0.45-μm pore size. The sulfadiazine, sulfamonomethoxine, and sulfamethoxazole concentrations were determined by UV–Vis.

### Statistical Analysis

Statistical analyses were conducted using Microcal Origin v. 7.5 (OriginLab Corp). The data obtained were analyzed for significant means using a one-way analysis of variance (ANOVA). Values were reported as means ± standard deviations, and statistical means were compared by *t-*test with *p* < 0.05.

## Results and Discussion

### Synthesis and Characterization of CD-g-CS

**Figure 1:**
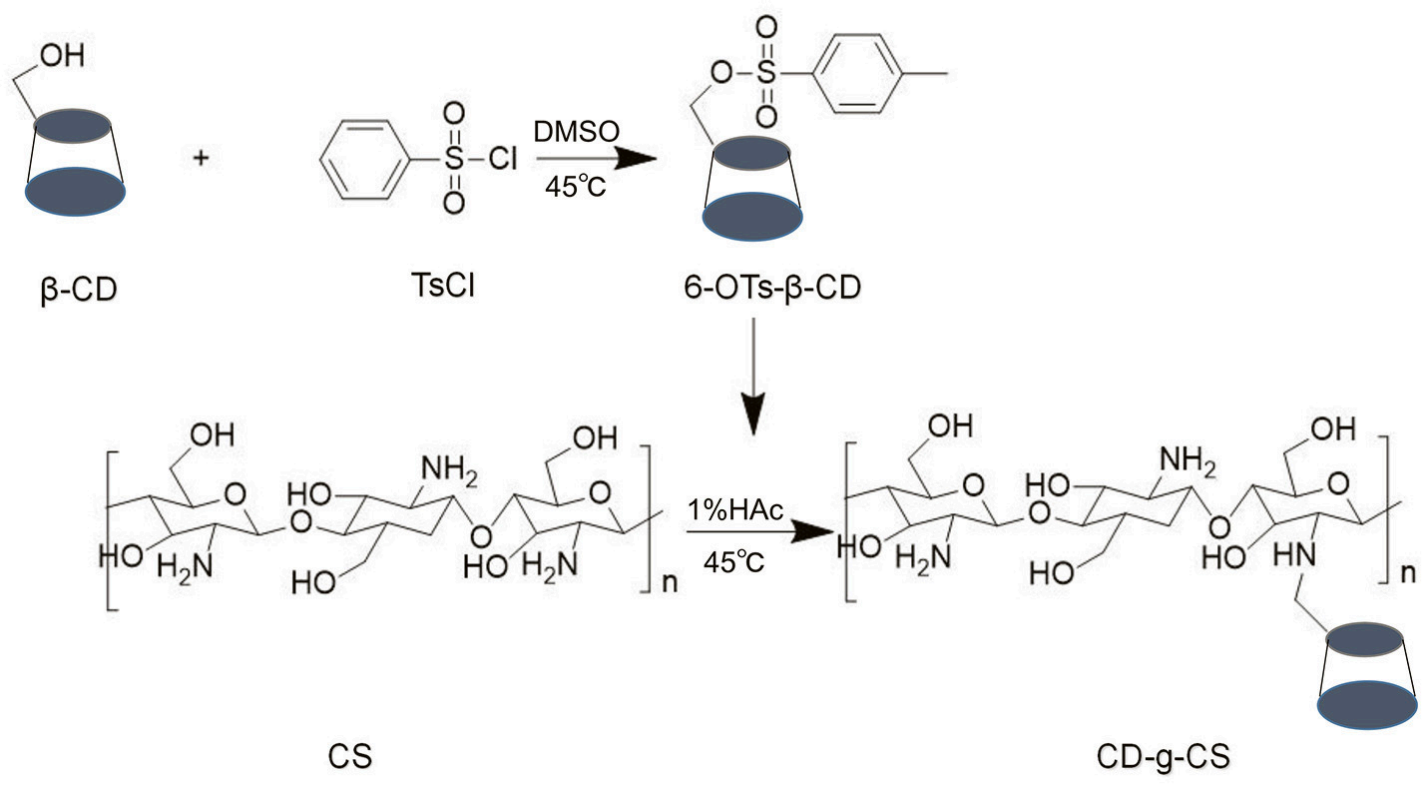
Synthetic pathway of CD-g-CS.

Generally, 6-OTs-β-CD is considered as the most widely used intermediate for changing the primary hydroxyl moiety of β-CD into other functional groups such as amino groups (Melton and Slessor, 1971). The resultant compound, 6-OTs-β-CD so formed is then grafted with the amino group in CS under acidic conditions (Figure 1). CD-g-CS is therefore the product of the reaction between 6-OTs-β-CD and CS in an acid-controlled environment. Thus, CS was protonated under this environment.

**Figure 2:**
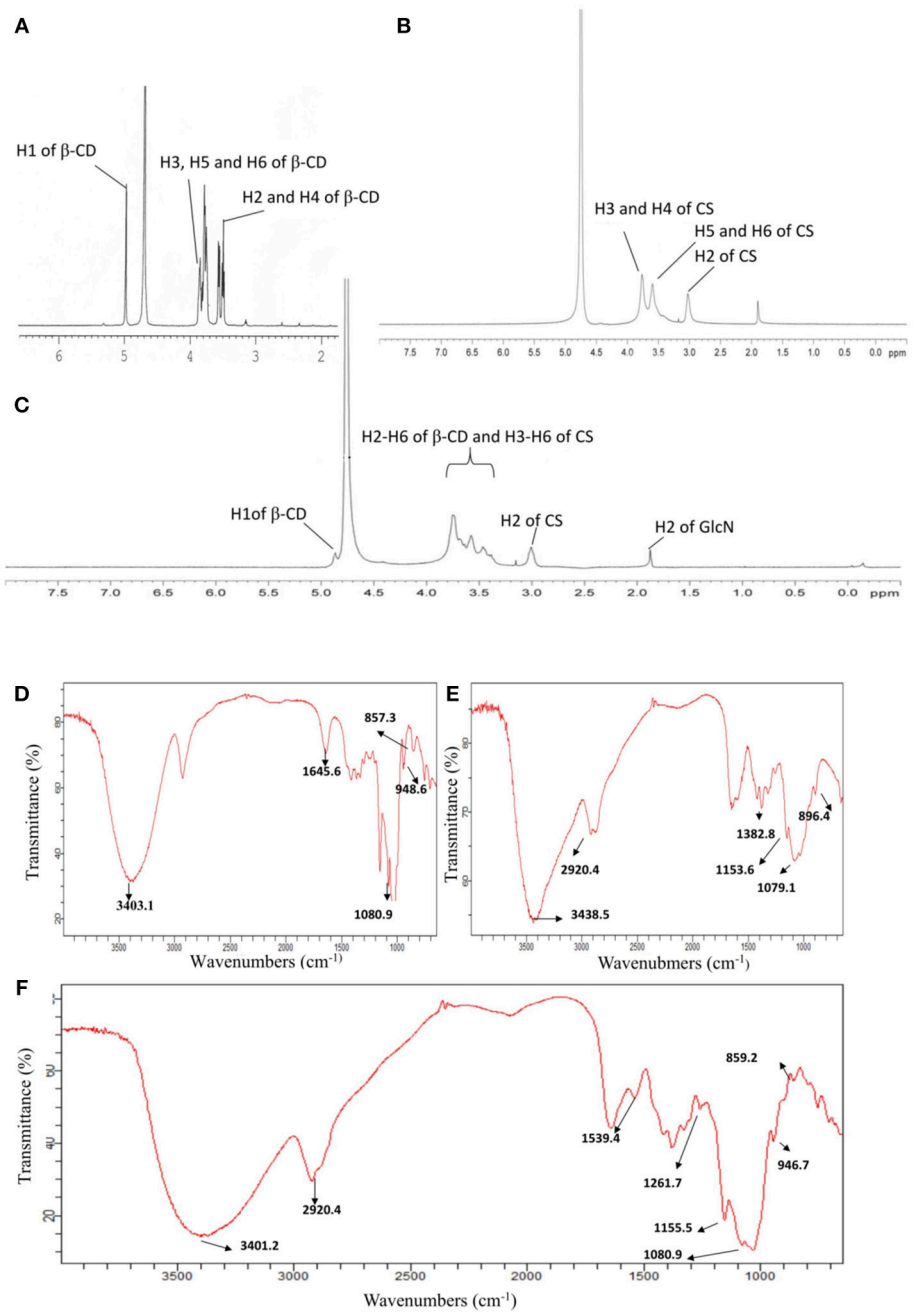
^1^H NMR spectra of β–CD **(A)**, CS **(B)**, and CD-g-CS **(C)**. FT-IR spectra of β-CD **(D)**, CS **(E)**, and CD-g-CS **(F)**.

The chemical structures of CS, β-CD and CD-g-CS were characterized by ^1^H NMR and FT-IR (see Figure 2). The ^1^H NMR spectrum of CS and β-CD is assigned in the Supplementary Material. In the ^1^H NMR spectrum of CD-g-CS (Figure 2C), the multiplet proton signals at δ = 5.0–3.0 ppm are attributed to the H2–H6 protons of β-CD and H3–H6 of CS. The complex multiplet proton signals is because the D-galactopyranose and β-(1, 4)-2-amino-2-deoxy-D-glucopyranose structural units of β-CD and CS are very similar (Sajomsang et al., 2012), this similarities cause the peaks of β-CD and CS to overlaps each other. Moreover, singlet at δ = 1.875 ppm is attributed to the H2 proton of the glucosamine (GlcN). The signals at δ = 4.864 and 3.003 ppm result from the H1 proton of β-CD and H2 of CS, respectively (Gonil et al., 2011; Sajomsang et al., 2012; Yuan et al., 2013). These results conflict with the observation of Gonil et al. (2011), who did not observe an aromatic proton signal at around δ = 7 ppm. This signifies that the tosyl group present in the CD-g-CS structure was removed.

The FT-IR spectrum of CS is shown in the Supplementary Material (Brugnerotto et al., 2001). In the FT-IR spectrum of CD-g-CS (Figure 2F), the dominant peaks at wavenumbers 3401.2 and 1384.7 cm^−1^ are attributed to the –OH stretching and –C–O stretching of the CS backbone, respectively. Comparing the spectra of β-CD and CD-g-CS, the peak of C–O– at 1080.9 cm^−1^ is blurred in the spectrum of CD-g-CS. Furthermore, the dominant peak of α-(1,4) glucopyranose in β-CD at 859.2 cm^−1^ and the peak corresponding to the α-pyranyl vibration of β-CD at 946.7 cm^−1^ are also observed. These findings are in agreement with the report of Wang et al. (2007). The ^1^H NMR and FT-IR results suggest the formation of CD-g-CS.

Q_CD_ was measured by the phenol-sulfuric acid method according to the number of 6-OTs-β-CD groups in each primary amino group of CS. The phenol-sulfuric acid method can be used for the quantitative colorimetric microdetermination of oligosaccharides and polysaccharides. The method is simple and sensitive (Dubois et al., 1956). We found that the system formed by CS, phenol and concentrated sulfuric acid was not absorbed at 490 nm after the above treatment. Therefore, it does not interfere with the determination of β-CD by this method. Good linearity was achieved in the hydrolysate of β-CD (Figure S1). The results revealed that the CD-g-CS with two different Q_CD_ (0.643 × 10^3^ and 0.6 × 10^2^ μmol/g) were successfully prepared. The higher molar ratio of 6-OTs-β-CD to primary amino groups of CS resulted in the higher Q_CD_.

### Amino Content of CD-g-CS

According to Kasaai (2009), DD can be obtained as the actual amino content of CS divided by the ideal amino content. DD is a key structural characteristic of CS on which other biological, physico-chemical, and mechanical properties depend. Hence, the amino content of CD-g-CS was determined by the acid-base titration method (Yi et al., 2006). The results revealed that the amino contents of CD-g-CS with Q_CD_ = 0.643 × 10^3^ and 0.6 × 10^2^ μmol/g were 6.34 ± 0.072 and 9.41 ± 0.055%, respectively. The ideal amino content is 9.94% when DD is 100% (Yi et al., 2006). The amino content of CD-g-CS decreased to some extent as the amino groups on CS reacted with β-CD to form CD-g-CS. Thus, the higher amino content of CD-g-CS (Q_CD_ = 0.6 × 10^2^ μmol/g) compared to CD-g-CS (Q_CD_ = 0.643 × 10^3^ μmol/g) was related to CS being linked to less β-CD.

### Antimicrobial Assessments

**Figure 3:**
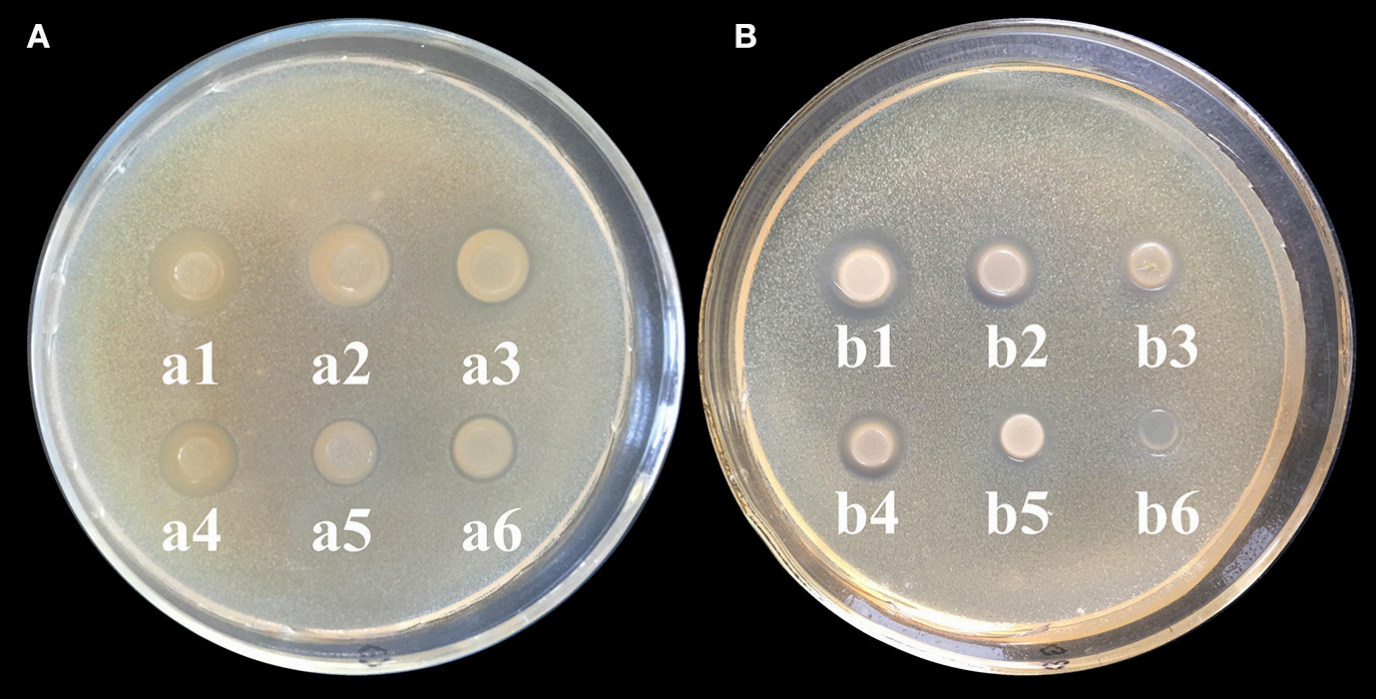
**(A,B)** Agar diffusion results for *S. xylosus* and *E. coli*, respectively. **a1 (b1)**, **a2 (b2)**, and **a3 (b3)** show the inhibition zones for 10, 5, and 2.5 mg/mL CD-g-CS (Q_CD_ = 0.6 × 10^2^ μmol/g), respectively. **a4 (b4)**, **a5 (b5)**, and **a6 (b6)** show the inhibition zones for 10, 5, and 2.5 mg/mL CD-g-CS (Q_CD_ = 0.643 × 10^3^ μmol/g), respectively.

CS and its derivatives have been reported to show some antimicrobial activity against microbial pathogens (Rabea et al., 2003). Thus, we investigated the antimicrobial activity of CD-g-CS to evaluate its potency as an antimicrobial excipient. In the present study, Gram-negative and Gram-positive bacteria were used to investigate of the antimicrobial activity of CD-g-CS. As shown in Figure 3A, the inhibition zone diameters for *S. xylosus* corresponding to 10, 5, and 2.5 mg/mL CD-g-CS (Q_CD_ = 0.6 × 1 0^2^ μmol/g) were 14.5 mm (**a1**), 13.5 mm (**a2**), and 12 mm (**a3**), respectively, and those for 10, 5, and 2.5 mg/mL CD-g-CS (Q_CD_ = 0.643 × 10^3^ μmol/g) were 12.5 mm (**a4**), 10 mm (**a5**), and 10 mm (**a6**), respectively. For *E. coli* (Figure 3B), the inhibition zone diameters corresponding to 10, 5, and 2.5 mg/mL of CD-g-CS (Q_CD_ = 0.6 × 10^2^ μmol/g) and CD-g-CS (Q_CD_ = 0.643 × 10^3^ μmol/g) were 13.5 mm (**b1**), 11.5 mm (**b2**), and 9.5 mm (**b3**), 11 mm (**b4**), 9 mm (**b5**), and 8 mm, and no inhibition zone **(b6**), respectively.

CD-g-CS exhibited antimicrobial activity against *S. xylosus* and *E. coli* as a result of the prominent antimicrobial properties of CS, as reported by Chen et al. (2017). Previous studies have also reported the antimicrobial activity of CS against bacteria (Hamdi et al., 2018). The difference in the antibacterial ability of CD-g-CS with Q_CD_ = 0.643 × 10^3^ and 0.6 × 10^2^ μmol/g may be attributed to the amount of CS. Studies have shown that sterilization with CS results in the elimination of pathogens. The concentration of CS and antibacterial activity are positively related (i.e., a higher CS concentration results in a stronger antimicrobial effect). The mechanism behind this effect can be explained as follows. As the concentration of CS increases, there is a resultant increase in the amount of free amino groups, leading to more positive charges under acidic conditions (Chen et al., 2002). The antimicrobial activity of the CS is based on the idea that the positive charge density of CS absorbed onto the negatively charged cell surfaces of bacteria leads to the leakage of proteinaceous and other intracellular constituents, causing cell death (Gonil et al., 2011). In this study, the amino content of CD-g-CS with Q_CD_ = 0.6 × 10^2^ μmol/g was greater than that of CD-g-CS with Q_CD_ = 0.643 × 10^3^ μmol/g, and the positive charge density was higher in determination of amino content of CD-g-CS. Thus, CD-g-CS with Q_CD_ = 0.6 × 10^2^ μmol/g showed better antibacterial activity.

### Effect of CD-g-CS on Bacterial Membrane Integrity

**Figure 4:**
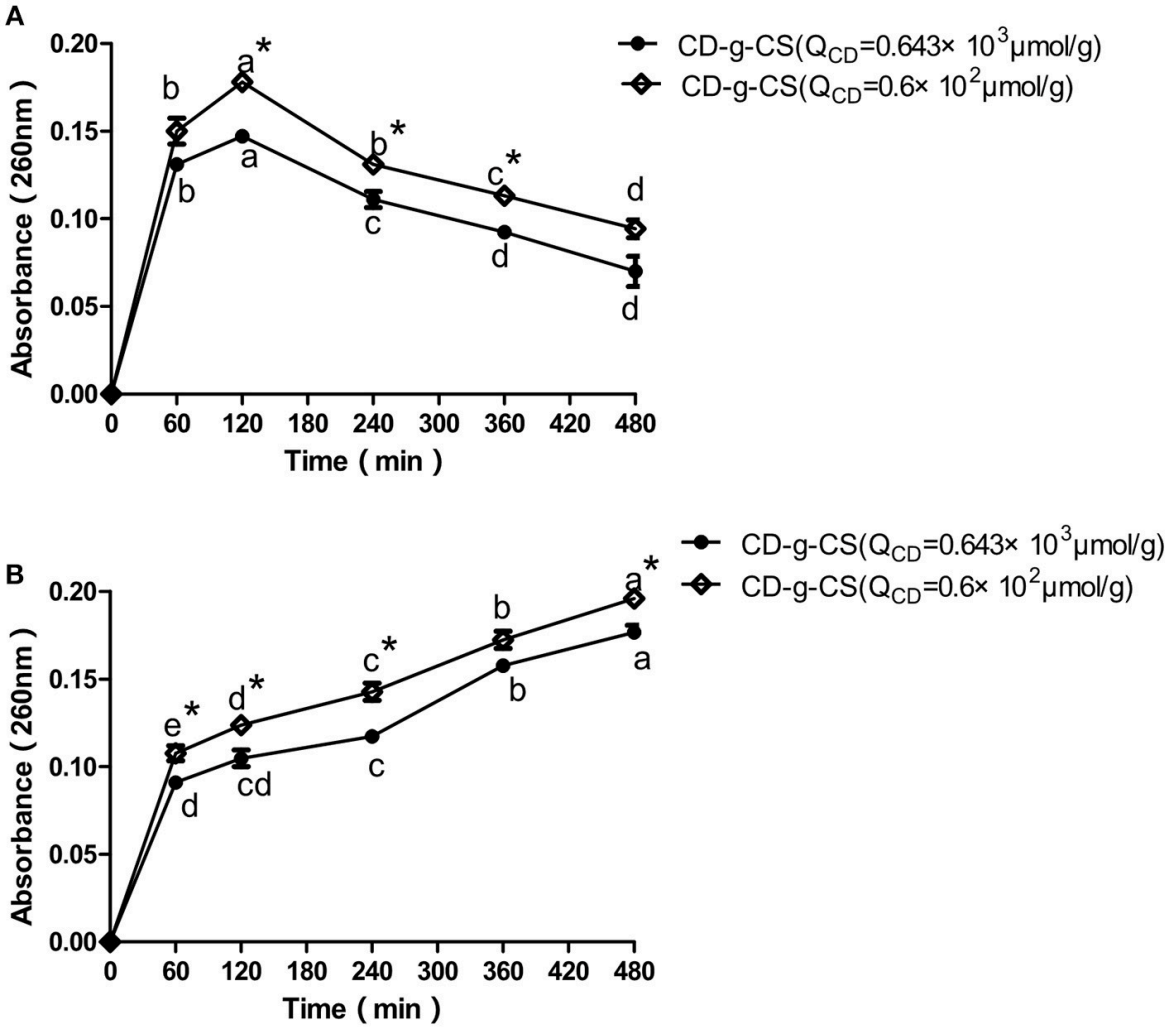
Release of intracellular components of *E. coli* **(A)** and *S. xylosus* **(B)** suspensions treated with 10 mg/mL of CD-g-CS (Q_CD_ = 0.643 × 10^3^ μmol/g) and CD-g-CS (Q_CD_ = 0.6 × 10^2^ μmol/g). Different letters indicate a significant difference within groups at *p* ≤ *0.05*. ^*^Indicates a significant difference between the control and CD-g-CS (Q_CD_ = 0.643 × 10^3^ μmol/g) at *p* ≤ 0.05. Data are expressed as mean ± standard deviation (*n* = 3).

The intracellular components of cells are protected by cell membranes. Thus, the release of the intracellular components of bacterial cells can be used to monitor the membrane integrity (Denyer and Hugo, 1991). The detection of DNA and RNA from the protoplasm of bacterial cells signifies that the cell membrane has been ruptured (Chen and Cooper, 2002). Thus, the amount of DNA and RNA detected reveals the extent of damage done to the bacterial cell. Figure 4 shows the amount of DNA and RNA released from *S. xylosus* and *E. coli* cells in suspensions treated with CD-g-CS. Initially, a rapid increase in the OD_260nm_ of *E. coli* was observed at 60 and 120 min followed by a gentle decrease for CD-g-CS (Q_CD_ = 0.643 × 10^3^ μmol/g) and CD-g-CS (Q_CD_ = 0.6 × 10^2^ μmol/g), respectively. This is because CS can combine with teichoic acid (Gram-positive bacteria) on the cell wall and also through the interaction with the cell membrane, thereby leading to the leakage of some intracellular substances (Tokura et al., 1997; Tsai and Su, 1999). Moreover, the thin and loosed crosslinking nature of the cell wall of Gram-negative bacteria may allow CD-g-CS to cause more damage to their cell walls. Thus, there was a rapid increase in the OD_260nm_ of *E. coli* up to 120 min. After that, the absorbance decreases may be adduced from the fact that the damage caused by CD-g-CS on *E. coli* reached its maximum value at 120 min. This was followed by the release of proteins or DNA gradually interacted CD-g-CS with the extension of time, resulting in the decrease of absorbance. Similar results were also reported for *Burkholderia seminalis* strain treated with CS. The result showed that the OD_260nm_ decreased with time after 60 min due to the interaction between CS and protein or DNA (Lou et al., 2011). For *S. xylosus* suspensions, the absorbance at 260 nm increased with time and depended on the amount of CD-g-CS. The release of intracellular components of *S. xylosus* and *E. coli* was greater for treatment with CD-g-CS (Q_CD_ = 0.6 × 10^2^ μmol/g) compared to CD-g-CS (Q_CD_ = 0.643 × 10^3^ μmol/g). This finding might be related to the fact that CS in CD-g-CS (Q_CD_ = 0.6 × 10^2^ μmol/g) was linked to a lower concentration of β-CD than in CD-g-CS (Q_CD_ = 0.643 × 10^3^ μmol/g), and the positive charge density of CD-g-CS (Q_CD_ = 0.6 × 10^2^ μmol/g) was higher. The CD-g-CS with high positive charge density absorbed onto the negatively charged cell surfaces of bacteria, leading to the leakage of intracellular constituents (Gonil et al., 2011). This is in line with the antimicrobial assessment tests. The results of this study indicate that CD-g-CS is bactericidal in nature and acts by rupturing the cell membranes of *S. xylosus and E. coli*, thereby causing the leakage of the DNA and RNA from the cells.

### The Effect of CD-g-CS on Microcosmic Morphology of Bacteria

**Figure 5:**
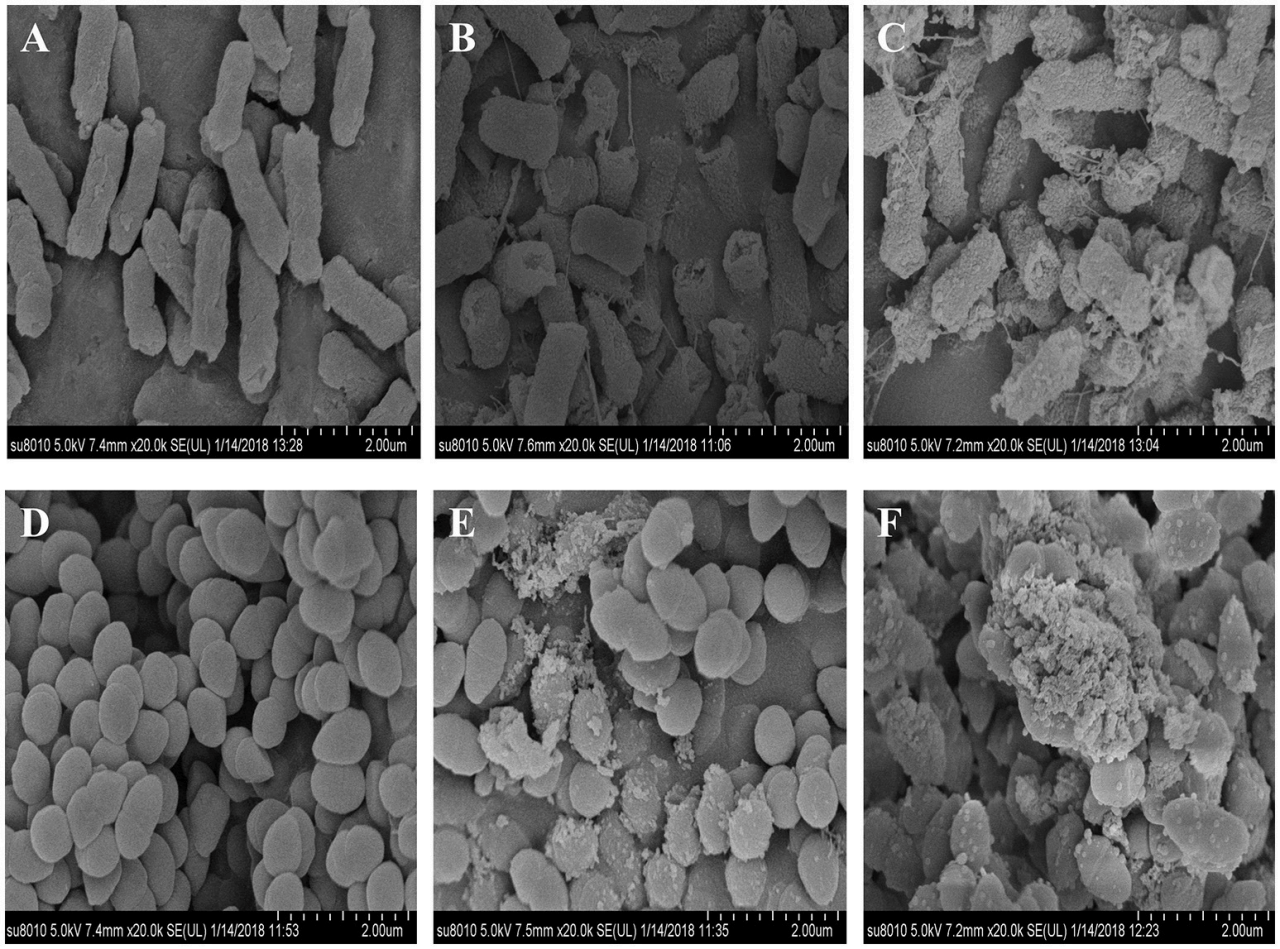
SEM micrographs of *E. coli* and *S. xylosus* untreated **(A,D)**, treated with 10 mg/mL CD-g-CS (Q_CD_ = 0.643 × 10^3^ μmol/g) **(B,E)**, and treated with 10 mg/mL CD-g-CS (Q_CD_ = 0.6 × 10^2^ μmol/g) **(C,F)**, respectively.

*E. coli* and *S. xylosus* untreated or treated with 10 mg/mL CD-g-CS were examined by SEM. Figures 5A,D show that in the controls *E. coli* and *S. xylosus* cells had intact cell membranes with electron-dense lines. On the other hand, *E. coli* treated with CD-g-CS showed disrupted and altered cell membrane after 1.5 h, especially in the groups treated with CD-g-CS (Q_CD_ = 0.6 × 10^2^ μmol/g). Figures 5B,C show that the CD-g-CS treated *E. coli* cells exhibited extracellular and intracellular changes compared to untreated cells. These changes included the following: the disruption of outer membrane structures with membrane sloughing and breaching; formation of irregular cell shapes and morphologies; degradation of bacilliform cells into short rods; formation of irregular condensed masses with bleb-like shapes; the development of faint bacterial profiles as a result of the loss of cell contents; blurring of the cell surface; and the development of dim, hollow and even crushed structures. The micrographs in Figure 5 showed that CD-g-CS damaged the cell membranes. Similar findings were reported by Helander et al. (2001) and Liu et al. (2004).

The micrographs of *S. xylosus* treated with CD-g-CS revealed severely damaged, atrophied, sunken and disrupted cell membranes after 1.5 h, especially the cells treated with CD-g-CS (Q_CD_ = 0.6 × 10^2^ μmol/g). *S. xylosus* cells were irregularly shaped, and the cell contents were lacking. In addition, the profiles of the bacteria became faint. Morphs of the bacteria were multiplied, and a small amount of cells were markedly elongated from the initial spherical shape. The results show that CD-g-CS affected the microcosmic morphology of *S. xylosus* (Figures 5D–F), as previously reported in an earlier study (Liu et al., 2004). Figure 5 shows that CD-g-CS (Q_CD_ = 0.6 × 10^2^ μmol/g) had a greater effect on the microcosmic morphology of bacteria than CD-g-CS (Q_CD_ = 0.643 × 10^3^ μmol/g). The result is in tandem with the effect of CD-g-CS on bacterial membrane integrity. The cell membrane integrity tests and SEM observation revealed that the antimicrobial mechanism of CD-g-CS was related to membrane disruption and cell lysis. The discovery of this antibacterial mechanism provides a scientific basis for the application of CD-g-CS as an antibacterial agent.

### Study of Drug Uptake Promoted by CD-g-CS in Bacteria

CS has previously been used to effectively enhance the absorption of hydrophilic drugs (Schipper et al., 1996). Nevertheless, the effect of CD-g-CS on drug uptake has not been reported. Thus, we used doxorubicin hydrochloride as a model drug to study the effect of CD-g-CS on drug uptake by *S. xylosus*. Doxorubicin hydrochloride exhibits red fluorescence under 480-nm excitation. After the bacterial uptake of doxorubicin hydrochloride, the amount of drug in the bacteria was indirectly reflected by the intensity of fluorescence.

**Figure 6:**
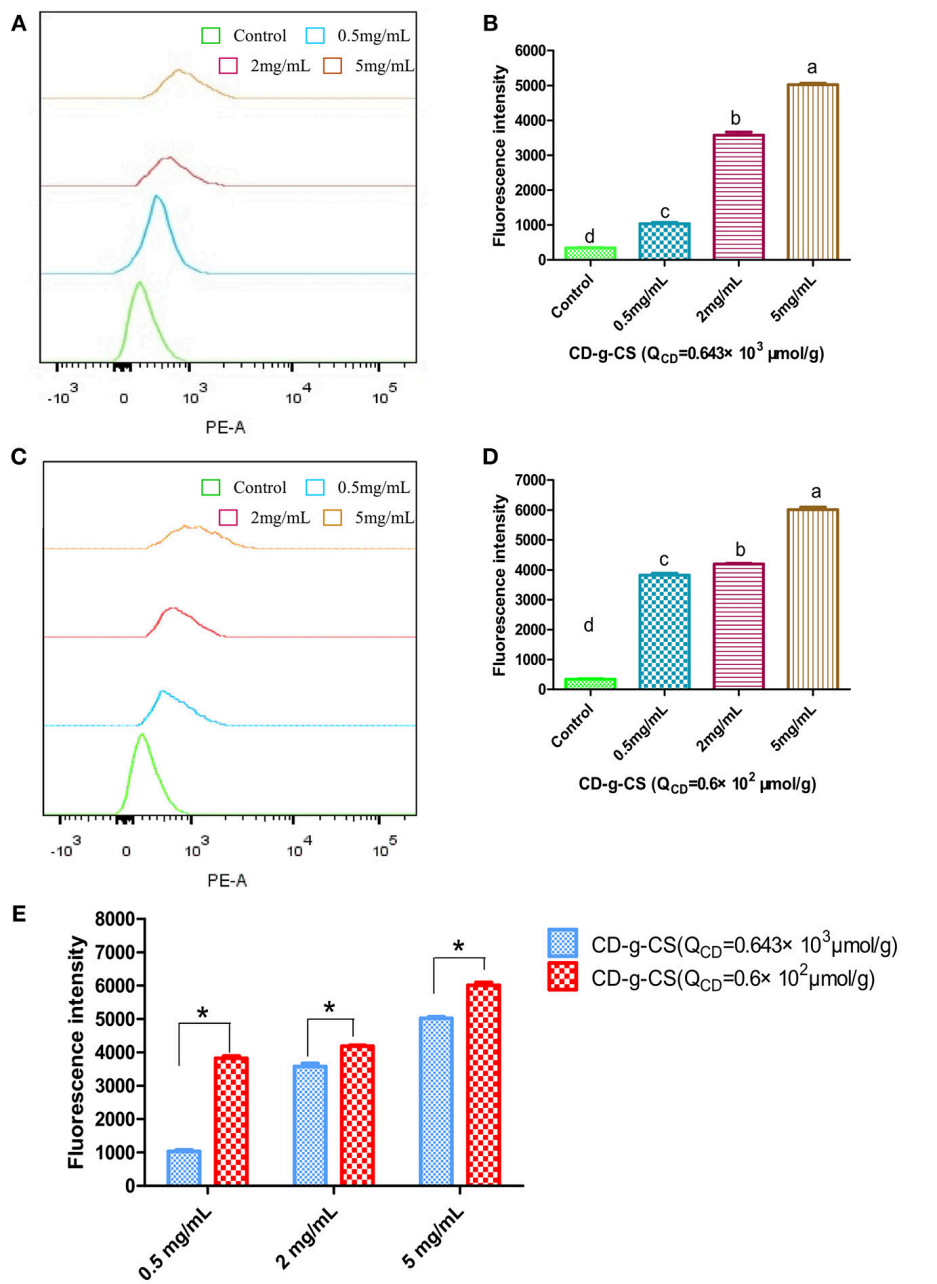
Quantitative *S. xylosus* uptake of doxorubicin hydrochloride by FCS. **(A,C)** Flueorescence intensity measured after *S. xylosus* was incubated with different concentrations of CD-g-CS (Q_CD_ = 0.643 × 10^3^ μmol/g) and CD-g-CS (Q_CD_ = 0.6 × 10^2^ μmol/g) (0.5, 2, and 5 mg/mL) for 1 h, respectively. **(B,D,E)** Fluorescence intensity measured at 0.5, 2, and 5 mg/mL, of CD-g-CS (Q_CD_ = 0.643 × 10^3^ μmol/g) and CD-g-CS (Q_CD_ = 0.6 × 10^2^ μmol/g), respectively (mean ± S.D, *n* = 3). Different letters indicate a significant difference within groups at *p* ≤ *0.05*. ^*^Indicates a significant difference between CD-g-CS (Q_CD_ = 0.643 × 10^3^ μmol/g) and CD-g-CS (Q_CD_ = 0.6 × 10^2^ μmol/g) at *p* ≤ 0.05.

The uptake of doxorubicin hydrochloride was investigated using FCS, as shown in Figure 6. The uptake of doxorubicin hydrochloride by *S. xylosus* was very low. However, with the extension of concentration, *S. xylosus* treated with CD-g-CS (Q_CD_ = 0.643 × 10^3^ μmol/g) and CD-g-CS (Q_CD_ = 0.6 × 10^2^ μmol/g). It was observed that the peak of fluorescence intensity shifted to the right compared to the control, and the concentration dependency was as pronounced as observed for the result, respectively. Namely, the fluorescence intensity of doxorubicin hydrochloride in *S. xylosus* increased significantly, especially for *S. xylosus* treated with CD-g-CS (Q_CD_ = 0.6 × 10^2^ μmol/g). The results demonstrate that CD-g-CS can promote the uptake of doxorubicin hydrochloride by *S. xylosus*. CD-g-CS (Q_CD_ = 0.6 × 10^2^ μmol/g) promoted uptake to a greater extent than CD-g-CS (Q_CD_ = 0.643 × 10^3^ μmol/g), and the uptake was concentration dependent.

**Figure 7:**
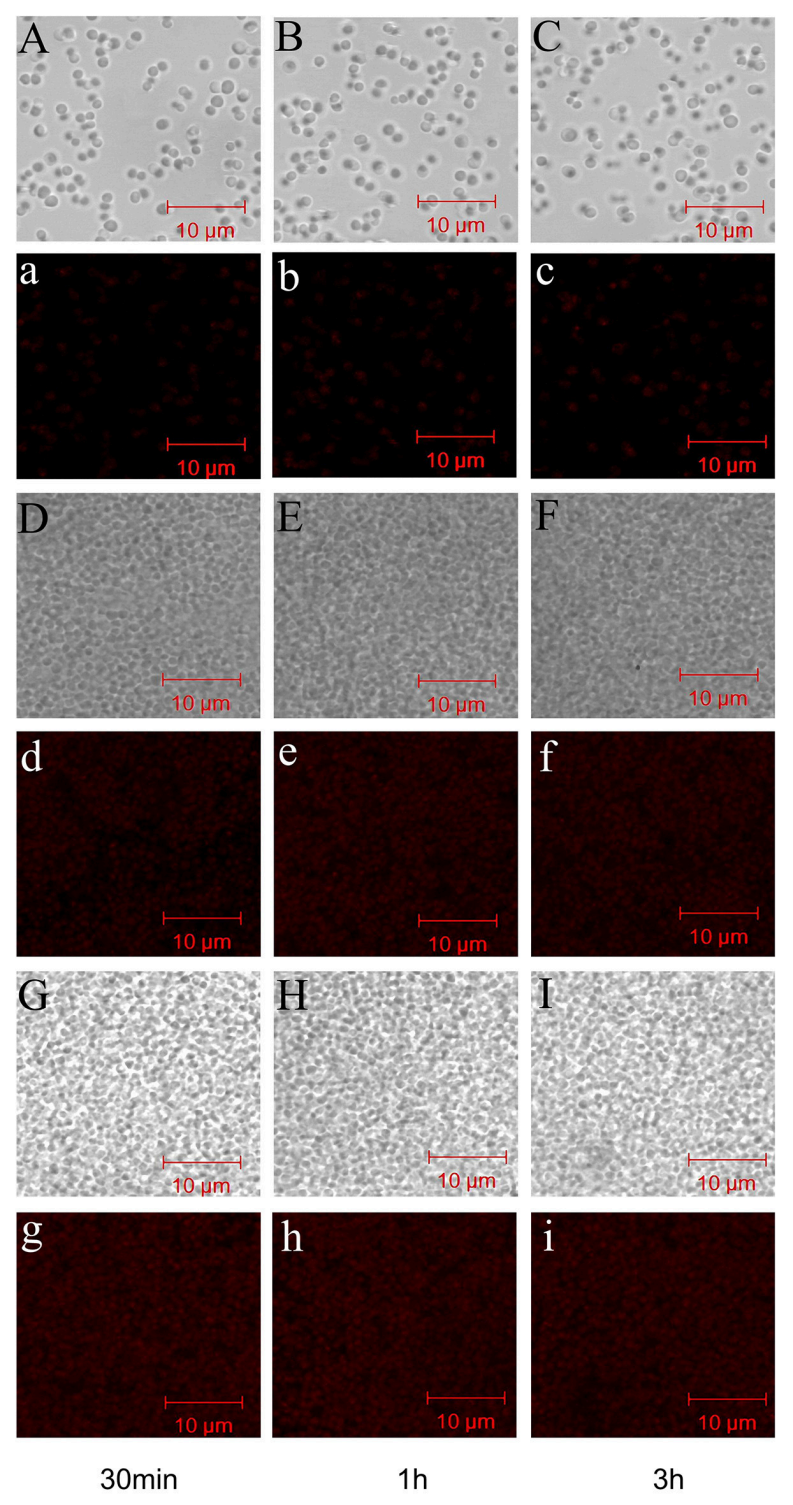
CLSM images showing the uptake of doxorubicin hydrochloride in *S. xylosus*. **(A–C)** Images of *S. xylosus* treated with only doxorubicin hydrochloride for 30 min, 1 and 3 h, respectively. **(D–I)** *S. xylosus* treated with CD-g-CS (Q_CD_ = 0.643 × 10^3^ μmol/g) and CD-g-CS (Q_CD_ = 0.6 × 1 0^2^ μmol/g) containing doxorubicin hydrochloride for 30 min, 1 and 3 h, respectively. **(a–i)** Bright-field CLSM images.

**Figure 8:**
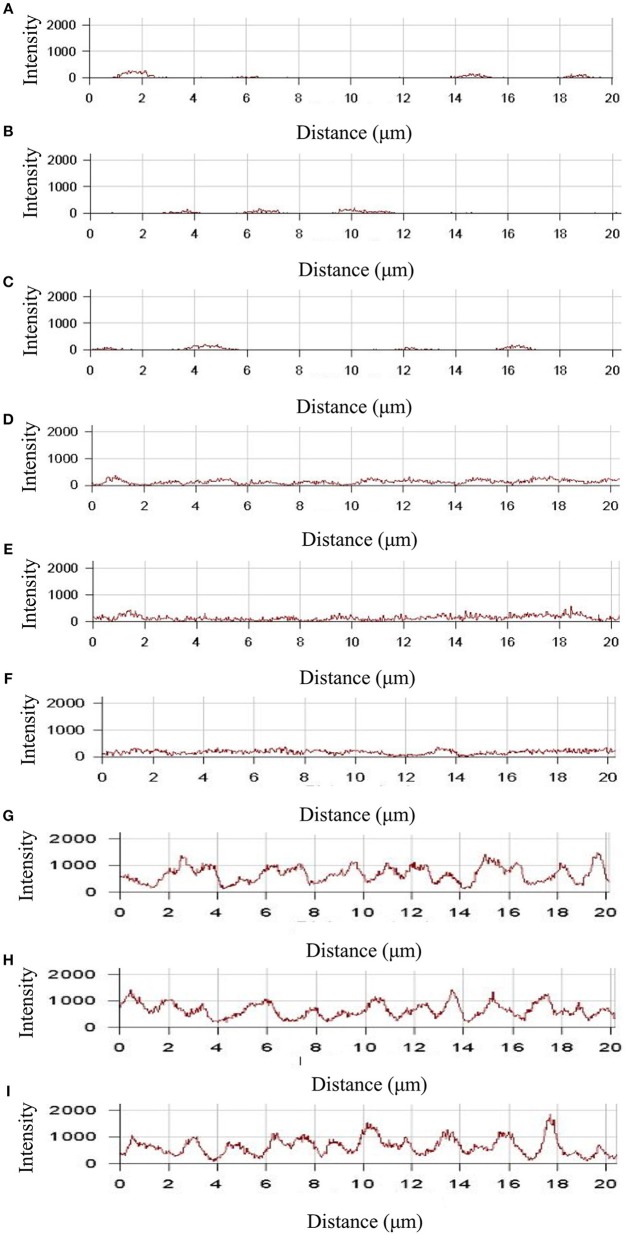
The fluorescence intensity was obtained by CLSM in *S. xylosus* uptake doxorubicin hydrochloride. **(A–C)** The fluorescence intensity of *S. xylosus* was treated with only doxorubicin hydrochloride for 30 min, 1 and 3 h, respectively. **(D–I)** *S. xylosus* was treated with CD-g-CS (Q_CD_ = 0.643 × 10^3^ μmol/g) and CD-g-CS (Q_CD_ = 0.6 × 10^2^ μmol/g) that contained doxorubicin hydrochloride for 30 min, 1 and 3 h, respectively.

To study the time dependence of drug uptake by bacteria treated with CD-g-CS, CLSM was used to observe the fluorescence intensity of bacteria. The results are presented in Figures 7, 8. In the CLSM images of the controls (Figures 7A–C, 8A–C), the fluorescence intensities of *S. xylosus* treated with only doxorubicin hydrochloride for 30 min, 1 and 3 h were very weak, and no significant differences were observed. With increasing incubation time for the same concentration of CD-g-CS, the fluorescence of *S. xylosus* was enhanced (Figures 7D–I). However, the fluorescence intensity was similar after *S. xylosus* treated with CD-g-CS (Q_CD_ = 0.643 × 10^3^ μmol/g) containing doxorubicin hydrochloride was incubated for 30 min, 1 and 3 h, respectively (Figures 8D–F). Similar results were found in the CD-g-CS (Q_CD_ = 0.6 × 10^2^ μmol/g) group (Figures 8G–I). Compared to the CD-g-CS (Q_CD_ = 0.643 × 10^3^ μmol/g) groups, the fluorescence intensity was higher in the CD-g-CS (Q_CD_ = 0.6 × 10^2^ μmol/g) groups, in agreement with the FCS results. Thus, we can conclude that CD-g-CS promotes the uptake of doxorubicin hydrochloride by *S. xylosus*, with CD-g-CS (Q_CD_ = 0.6 × 10^2^ μmol/g) showing a better promotion effect than CD-g-CS (Q_CD_ = 0.643 × 10^3^ μmol/g). However, the uptake was not time dependent.

CD-g-CS can promote the uptake of drugs by bacteria because it has the properties of CS (Chen et al., 2017). An earlier study by Illum et al. (1994) found that the absorption of peptides such as insulin and calcitonin across the nasal epithelium of rats and sheep was significantly enhanced by CS. Schipper investigated the effect of CS on poorly absorbable drugs across human epithelial cells and found that CS effectively enhanced the absorption of hydrophilic drugs such as peptides and proteins across nasal and intestinal epithelia (Schipper et al., 1996). Therefore, the mechanisms by which CD-g-CS and CS derivatives promote drug uptake may be the same because CS derivatives can increase membrane permeability (Je and Kim, 2006). The discovery that CD-g-CS can promote the uptake of drugs by bacteria is of great importance for improving the efficacy of antibiotics, reducing antibiotic dosage, and reducing antibiotic residues in animal-derived foods.

### The Solubilization Effect of CD-g-CS on Sulfonamides

**Figure 9:**
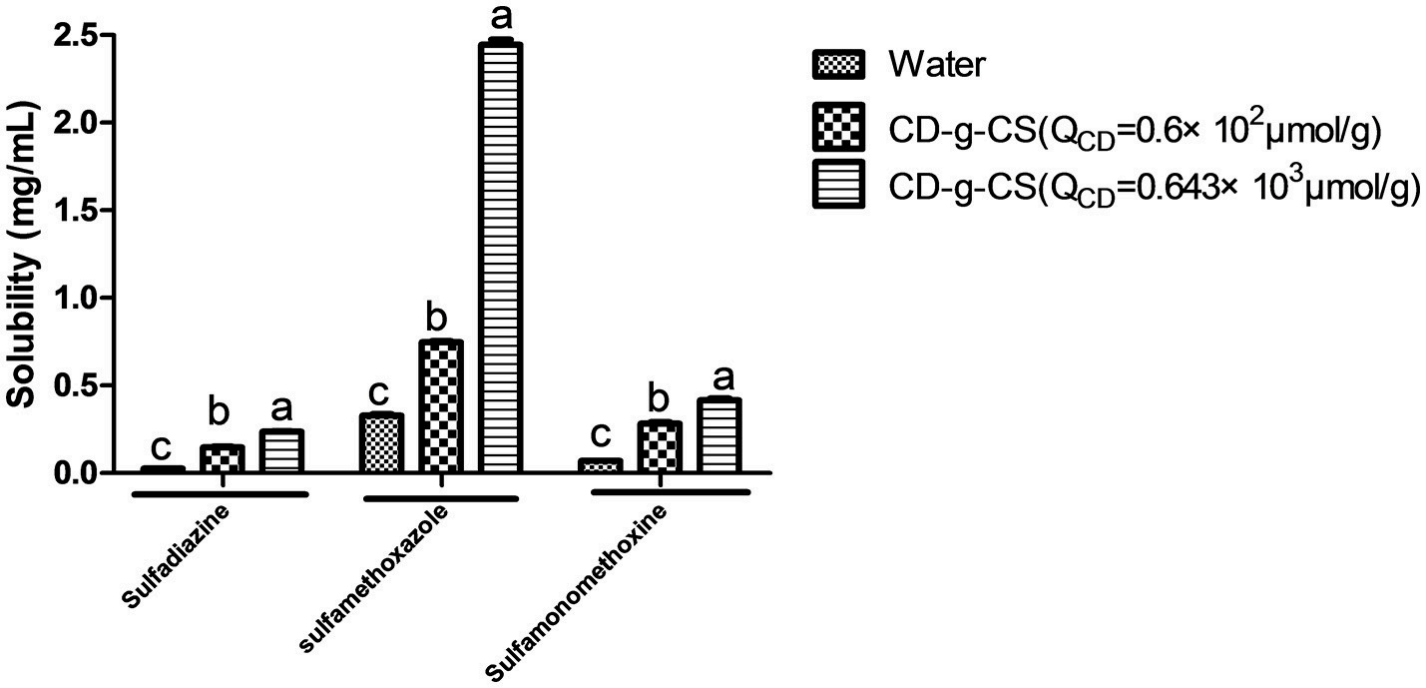
Effect of CD-g-CS on the solubility of sulfadiazine, sulfamonomethoxine and sulfamethoxazole. Different letters indicate a significant difference at *p* ≤ 0.05. Data are expressed as mean ± standard deviation (*n* = 3).

The inclusion complexes of poorly water-soluble drugs with CD-g-CS were investigated using sulfadiazine, sulfamonomethoxine, and sulfamethoxazole as model drugs. The methodological verification of sulfamethoxazole, sulfadiazine and sulfamonomethoxine was implemented by UV–Vis, such as linear, precision, stability, and recovery in Supplementary Material. The solubilities of sulfadiazine, sulfamonomethoxine, and sulfamethoxazole with CD-g-CS in water were studied, as shown Figure 9. The solubilities of sulfadiazine, sulfamonomethoxine, and sulfamethoxazole in water were 0.026 ± 0.00168, 0.069 ± 0.00159, and 0.329 ± 0.01157 mg/mL, respectively. However, the solubilities of sulfadiazine, sulfamonomethoxine and sulfamethoxazole with 10 mg/mL CD-g-CS (Q_CD_ = 0.6 × 10^2^ μmol/g) were 0.147 ± 0.00705, 0.283 ± 0.013, and 0.747 ± 0.00985 mg/mL, respectively, representing solubility enhancements of approximately 5.6, 4.1, and 2.3 times, respectively, compared to the aqueous solutions.

The respective solubilities of sulfadiazine, sulfamonomethoxine, and sulfamethoxazole with 10 mg/mL CD-g-CS (Q_CD_ = 0.643 × 10^3^ μmol/g) were 0.236 ± 0.00425, 0.416 ± 0.019, and 2.444 ± 0.05012 mg/mL, corresponding to enhancements of 9.1, 6.7, and 7.3 times, respectively. These results suggest that CD-g-CS increased the solubilities of sulfadiazine, sulfamonomethoxine, and sulfamethoxazole. This effect is attributed to the structure of the β-CD molecules of CD-g-CS. These molecules have a truncated cone structure with the ring having an interior hydrophobic cavity and an outer hydrophilic character. The hydrophobic interior and hydrophilic exterior allow β-CD to serve as a carrier to enhance the solubility of drugs that are poorly soluble in water. The addition of drug molecules into the hydrophobic cavity of β-CD improves the aqueous solubility, dissolution rate, bioavailability and stability of the drug (Szejtli, 1988). CD-g-CS can increase the solubility of sulfonamides, providing a new strategy for increasing the solubility of insoluble antibiotics. This finding is expected to facilitate the application of insoluble antibiotics in clinical treatment.

## Conclusions

CD-g-CS with two different Q_CD_ was successfully synthesized. CD-g-CS showed antimicrobial activity against *E. coli* and *S. xylosus*, and the antimicrobial activity of CD-g-CS was higher for the lower Q_CD_. Furthermore, the antimicrobial mechanism of CD-g-CS was due to membrane disruption and cell lysis based on cell membrane integrity tests and SEM observation. The uptake of doxorubicin hydrochloride in *S. xylosus* was significantly increased by CD-g-CS, demonstrating that CD-g-CS can promote the uptake of drugs by bacteria. In addition, CD-g-CS can enhance the solubility of sulfadiazine, sulfamonomethoxine, and sulfamethoxazole. The findings of this study can help reduce the use of antibiotics and suggest new ways to solve the problems caused by antibiotics in clinical application (e.g., bacterial resistance and antibiotic residues in animal-derived foods).

## Author Contributions

The experiment was designed by Y-HL and directed by W-YD and S-DZ. YQ, FY, J-WB, W-QC, TY, X-RC, and GB-O were supportive during the experiment.

### Conflict of Interest Statement

The authors declare that the research was conducted in the absence of any commercial or financial relationships that could be construed as a potential conflict of interest.
