# Supplementary material for: Chitosan Grafted With β-Cyclodextrin: Synthesis, Characterization, Antimicrobial Activity, and Role as Absorbefacient and Solubilizer
Source: Front Chem. 2019 Jan 10;6:657. doi: 10.3389/fchem.2018.00657 (PMC6335354; doi:10.3389/fchem.2018.00657)
Supplement: Supplementary file 1 [file Table_1.doc]

***Supplementary Material***

Chitosan Grafted with β-Cyclodextrin: Synthesis, Characterization, Antimicrobial Activity, and Role as Absorbefacient and Solubilizer

*Wen-Ya* *Ding^1,2,3+^, Si-Di Zheng^1,2+^, Yue Qin^1,2^, Fei Yu^1,2^, Jing-Wen Bai^4^, Wen-Qiang Cui^1,2^, Tao Yu^1,2^, Xing-Ru Chen^1,2^, Bello-Onaghise God'spower^1,2^, Yan-Hua Li^1,2*^*

*1College of Veterinary Medicine, Northeast Agricultural University, Harbin, Heilongjiang 150030*

*2Heilongjiang Key Laboratory for Animal Disease Control and Pharmaceutical Development, Harbin, China*

*3Northeastern Science Inspection Station, China Ministry of Agriculture Key Laboratory of Animal Pathogen Biology, Northeast Agricultural University, Harbin, Heilongjiang 150030, China*

*4College of Science, Northeast Agricultural University, Harbin, China*

*Correspondence to: Professor Yanhua Li, College of Veterinary Medicine, Northeast Agricultural University, 600 Changjiang Road, Xiangfang, Harbin, Heilongjiang 150030, P.R. China

Tel：+86 451 55191881

E‑mail: liyanhua1970@163.com (Y.-H.Li).

^†^These authors have contributed equally to this study and share first authorship.

The characterization of the chemical structures of CS and β-CD

**Figure 2A** showed the ^1^H NMR spectrum of the β-CD. The proton signals at δ 4.9 ppm and the multiplet proton signals at δ 3.9–3.4 ppm, which were due to the H1 proton and H2–H6 protons, respectively (Gonil et al., 2011). According to Ben’s report (Ben Mihoub et al., 2018), the protons were further assigned as follows: The multiplet protons at δ=3.9–3.7 ppm were assigned to the H3, H5 and H6 protons. The multiplet protons at δ=3.6–3.4 ppm were assigned to the H2, H4 protons. The ^1^H NMR of CS is shown in **Figure 2B**. ^1^H NMR (DCl) δ=3.025 (H2) (Zhu et al., 2006). Due to the similar chemical environments and chemical shift of H3, H4, H5 and H6, the peaks covered each other. It has been reported that the H3 and H4 chemical shift of glucosamine are before H5 and H6 (Cui et al., 2017). Thus δ=3.764 ppm and 3.597 ppm resulted from the H3, H4 proton and H5, H6, respectively. The H1 chemical shift of chitosan is usually 4.4 to 5.0 and overlaps with solvent peak (And and Rinaudo, 2001).

The FT-IR of CS (**Figure 2E**) showed a broad -OH stretch spectrum at 3438.5 cm^−1^ and the C-H chain stretch within the 2990 and 2850 cm^−1^ range. The free primary amino group at C2 position was represented by the other major absorption within the 1220 and 1020 cm^−1^ region. The –C–O stretch of primary alcoholic group was observed at peak 1382.8 cm^−1^.

**The standard curve of hydrolysis of β-CD**


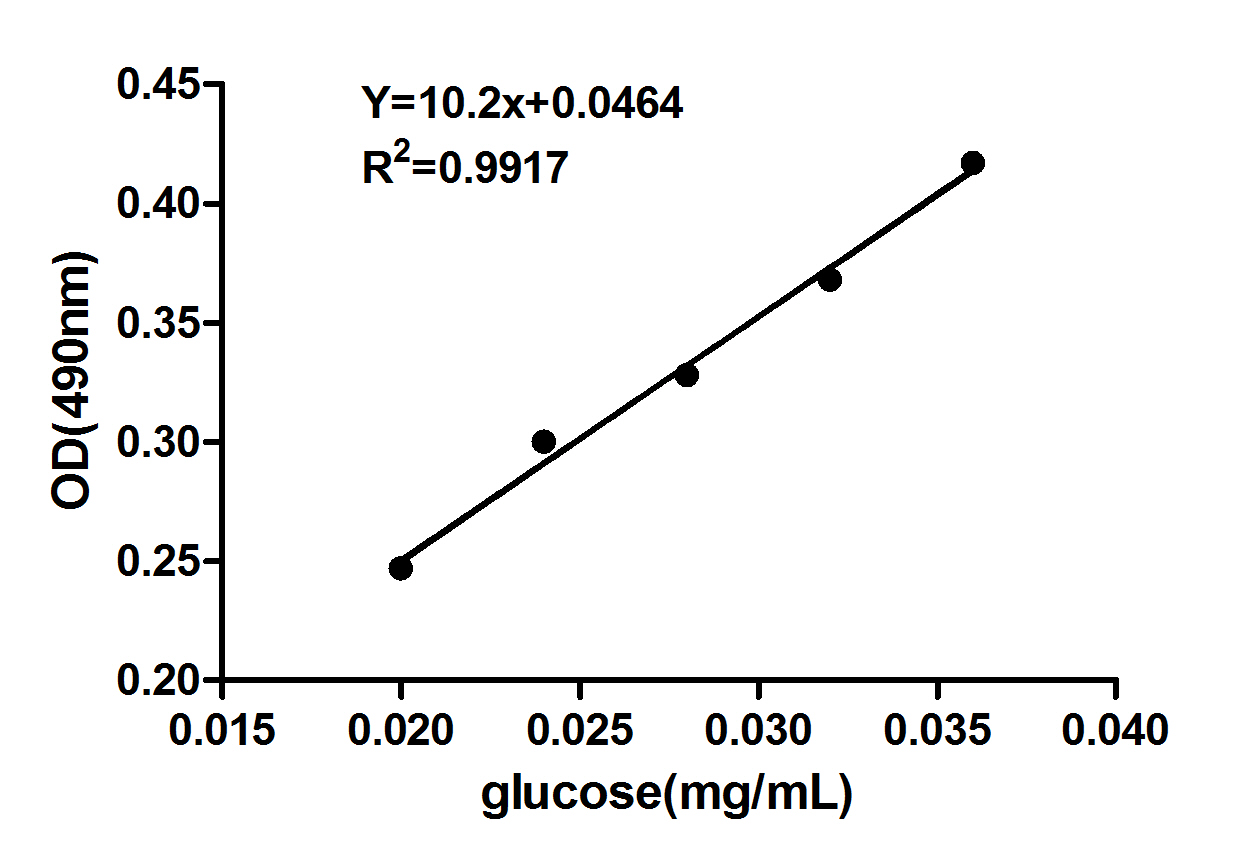


Fig. S1. The standard curve of hydrolysis of β-CD.

**Linearity**

The calibration curve prepared by plotting the absorbance versus concentration of [sulfadiazine](javascript:;) and [sulfamonomethoxine](javascript:;) from 4 to 8 μg/mL, the concentration of [sulfamethoxazole](javascript:;) were 4 μg/mL, 6 μg/mL, 8 μg/mL, 10 μg/mL and 12 μg/mL, respectively, consisting of five concentrations analyzed in solutions of authentic standards; the calibration curve was the average of five authentic standard curves. The [maximum](javascript:;) [absorption](javascript:;) [wavelength](javascript:;) of [sulfadiazine](javascript:;), [sulfamonomethoxine](javascript:;) and [sulfamethoxazole](javascript:;) were 254nm, 262nm and 257nm, which was determined after scanning the wavelength range of the UV–Vis spectrophotometer. The linear equation of [sulfadiazine](javascript:;), [sulfamonomethoxine](javascript:;) and [sulfamethoxazole](javascript:;) were $\text{y}=0.0913\text{x}-0.0202$, $\text{y}=0.085\text{x}-0.0072$ and $\text{y}=0.0609\text{x}+0.0028$ and the linear correlation coefficient (R^2^) were 0.9995, 0.9987 and 0.9981, respectively.

**Precision**

The precision was determined by analyzing [sulfadiazine](javascript:;), [sulfamonomethoxine](javascript:;) and [sulfamethoxazole](javascript:;) of known concentrations at three levels. The solutions of [sulfadiazine](javascript:;) and [sulfamonomethoxine](javascript:;) were prepared in 0.1mol/LNaOH and [sulfamethoxazole](javascript:;) was prepared in [absolute](javascript:;) [ethyl](javascript:;) [alcohol](javascript:;) with concentrations at the minimum, intermediate and maximum linearity points defined in the linearity test. The solutions and samples were analyzed in triplicate on different days, and the concentrations were recalculated each time using the calibration curve obtained for the UV–Vis method. Table. S1 presents the precision results used to validate the UV–Vis method presented as an average of triplicate measurements on different days ± the standard deviation and the associated %RSD. The precision evaluation of the proposed method yielded RSDs below 2% at all evaluated levels and the precision was satisfactory.

Table. S1. Precision evaluation for the analytical validation of the UV–Vis method for [sulfadiazine](javascript:;), [sulfamonomethoxine](javascript:;) and [sulfamethoxazole](javascript:;) quantification (n = 3).

| Sample | Add Concentration（μg/mL） | Day | Intra-day precision | | Inter-day precision | |
| --- | --- | --- | --- | --- | --- | --- |
|  |  |  | Measured concentration（μg/mL） | RSD（%） | Measured concentration（μg/mL） | RSD（%） |
| [sulfadiazine](javascript:;) | 4 | 1^st^ day | 3.85±0.04 | 0.91 | 3.91±0.05 | 1.36 |
|  |  | 2^nd^ day | 3.95±0.02 | 0.42 |  |  |
|  |  | 3^rd^ day | 3.95±0.02 | 0.56 |  |  |
|  | 6 | 1^st^ day | 5.91±0.02 | 0.28 | 5.86±0.09 | 1.53 |
|  |  | 2^nd^ day | 5.75±0.07 | 1.19 |  |  |
|  |  | 3^rd^ day | 5.92±0.03 | 0.47 |  |  |
|  | 8 | 1^st^ day | 7.99±0.04 | 0.55 | 7.96±0.06 | 0.71 |
|  |  | 2^nd^ day | 7.90±0.02 | 0.28 |  |  |
|  |  | 3^rd^ day | 8.01±0.02 | 0.28 |  |  |
| [sulfamonomethoxine](javascript:;) | 4 | 1^st^ day | 4.01±0.04 | 0.90 | 4.13±0.06 | 1.56 |
|  |  | 2^nd^ day | 3.98±0.03 | 0.78 |  |  |
|  |  | 3^rd^ day | 3.94±0.06 | 1.64 |  |  |
|  | 6 | 1^st^ day | 5.95±0.03 | 0.43 | 5.99±0.05 | 0.88 |
|  |  | 2^nd^ day | 5.97±0.02 | 0.41 |  |  |
|  |  | 3^rd^ day | 6.04±0.05 | 0.81 |  |  |
|  | 8 | 1^st^ day | 11.58±0.08 | 0.65 | 7.91±0.08 | 0.95 |
|  |  | 2^nd^ day | 11.90±0.07 | 0.56 |  |  |
|  |  | 3^rd^ day | 11.71±0.07 | 0.57 |  |  |
| [sulfamethoxazole](javascript:;) | 4 | 1^st^ day | 3.83±0.06 | 1.55 | 4.13±0.06 | 1.56 |
|  |  | 2^nd^ day | 3.90±0.03 | 0.64 |  |  |
|  |  | 3^rd^ day | 3.94±0.05 | 1.23 |  |  |
|  | 8 | 1^st^ day | 7.96±0.03 | 0.43 | 8.48±0.12 | 1.39 |
|  |  | 2^nd^ day | 8.11±0.05 | 0.62 |  |  |
|  |  | 3^rd^ day | 7.90±0.13 | 1.62 |  |  |
|  | 12 | 1^st^ day | 11.58±0.08 | 0.65 | 12.44±0.15 | 1.21 |
|  |  | 2^nd^ day | 11.90±0.07 | 0.56 |  |  |
|  |  | 3^rd^ day | 11.71±0.07 | 0.57 |  |  |

**Stability**

The stability were determined that three replicates of the quality control samples at 6μg/mL of [sulfadiazine](javascript:;), [sulfamonomethoxine](javascript:;) and 8μg/mL of [sulfamethoxazole](javascript:;) were processed and analyzed according to requirements, respectively. The stability test was performed by keeping the samples for 0h, 1h, 2h, 4h, 6h, 8h, 24h at room temperature before analysis. The stability of [sulfadiazine](javascript:;), [sulfamonomethoxine](javascript:;) and [sulfamethoxazole](javascript:;) are shown in the Table. S2. This result indicated that [sulfadiazine](javascript:;), [sulfamonomethoxine](javascript:;) and [sulfamethoxazole](javascript:;) were stable at room temperature for 24h.

Table. S2. The stability evaluation of the UV–Vis method for [sulfadiazine](javascript:;), [sulfamonomethoxine](javascript:;) and [sulfamethoxazole](javascript:;) at room temperature (n = 3).

| Sample | Add Concentration（μg/mL） | Measured concentration（μg/mL） | | | | | | | RSD  (%) |
| --- | --- | --- | --- | --- | --- | --- | --- | --- | --- |
|  |  | 0h | 1h | 2h | 4h | 6h | 8h | 24h |  |
| [sulfadiazine](javascript:;) | 6 | 5.98±  0.078 | 6.04±  0.058 | 6.13±  0.013 | 5.89±  0.047 | 5.95±  0.055 | 6.14±  0.043 | 6.02±  0.054 | 1.95 |
| [sulfamonomethoxine](javascript:;) | 6 | 6.09±  0.060 | 6.09±  0.026 | 5.93±  0.021 | 6.12±  0.018 | 5.88±  0.018 | 6.12±  0.046 | 6.13±  0.015 | 1.8 |
| [sulfamethoxazole](javascript:;) | 8 | 7.97±  0.046 | 7.88±  0.052 | 8.17±  0.019 | 8.19±  0.023 | 8.23±  0.033 | 8.06±  0.161 | 8.05±  0.047 | 1.95 |

**Recovery**

The percentage recovery was determined by analyzing [sulfadiazine](javascript:;) and [sulfamonomethoxine](javascript:;) at concentrations of 6μg/mL, [sulfamethoxazole](javascript:;) of 8μg/mL respectively. Since no certified reference material existed, 6.061µg/mL [sulfadiazine](javascript:;) and [sulfamonomethoxine](javascript:;) were added to 6μg/mL, 8.108µg/mL [sulfamethoxazole](javascript:;) were added to 8μg/mL. The solutions and samples were analyzed in triplicate, and the concentrations were recalculated each time using the calibration curve obtained for the UV–Vis method. From Table. S3, We obtained average recoveries above 98%, with RSDs below 2% at all levels investigated. These results showing that instrumental and reagent manufacturer variations did not influence the [sulfadiazine](javascript:;), [sulfamonomethoxine](javascript:;) and [sulfamethoxazole](javascript:;) quantification.

Table. S3. Recovery evaluation for the analytical validation of the UV–Vis method for [sulfadiazine](javascript:;), [sulfamonomethoxine](javascript:;) and [sulfamethoxazole](javascript:;) quantification.

| Sample | Concentration（μg/mL） | Add Concentration（μg/mL） | Recovery rate mean (%) | RSD(%) |
| --- | --- | --- | --- | --- |
| [sulfadiazine](javascript:;) | 6 | 6.061 | 98.98±0.21 | 0.88 |
| [sulfamonomethoxine](javascript:;) | 6 | 6.061 | 99.5±0.67 | 0.64 |
| [sulfamethoxazole](javascript:;) | 8 | 8.108 | 99.2±0.23 | 0.76 |

Reference

And, R.A., and Rinaudo, M. (2001). Chitosan Derivatives Bearing Pendant Cyclodextrin Cavities: Synthesis and Inclusion Performance. *Macromolecules* 34(11)**,** 3574-3580.

Ben Mihoub, A., Saidat, B., Bal, Y., Frochot, C., Vanderesse, R., and Acherar, S. (2018). Development of new ionic gelation strategy: Towards the preparation of new monodisperse and stable hyaluronic acid/beta-cyclodextrin-grafted chitosan nanoparticles as drug delivery carriers for doxorubicin. *Frontiers of Materials Science* 12(1)**,** 83-94. doi: 10.1007/s11706-018-0407-2.

Cui, G.H., Zhao, D.H., Lv, P., Gao, Z.G., Chen, S.Y., Qiu, N.N., et al. (2017). Synthesis and characterization of Eu(III)-based coordination complexes of modified D-glucosamine and poly(N-isopropylacrylamide). Optical Materials 72, 115-121. doi: 10.1016/j.optmat.2017.05.051.

Gonil, P., Sajomsang, W., Ruktanonchai, U.R., Pimpha, N., Sramala, I., Nuchuchua, O., et al. (2011). Novel quaternized chitosan containing β-cyclodextrin moiety: Synthesis, characterization and antimicrobial activity. *Carbohydrate Polymers* 83(2)**,** 905-913.

Zhu, A., Chen, T., Yuan, L., Wu, H., and Lu, P. (2006). Synthesis and characterization of N-succinyl-chitosan and its self-assembly of nanospheres. *Carbohydrate Polymers* 66(2)**,** 274-279. doi: 10.1016/j.carbpol.2006.03.014.
